# Supplementary figures and images for: Clinical management and genomic profiling of pediatric low-grade gliomas in Saudi Arabia
Source: PLoS One. 2020 Jan 29;15(1):e0228356. doi: 10.1371/journal.pone.0228356 (PMC6988947; doi:10.1371/journal.pone.0228356)

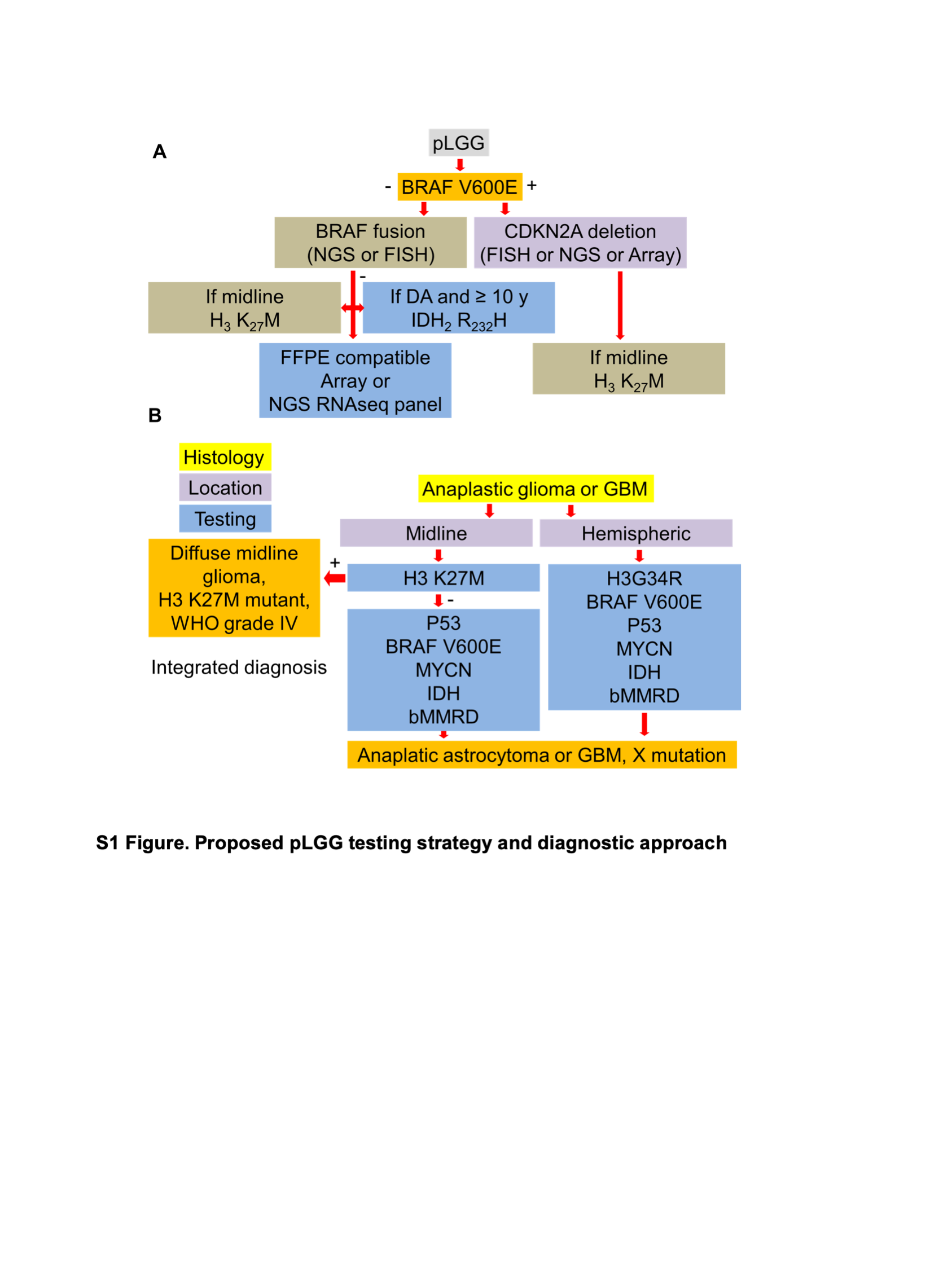

Supplement: S1 Fig — (TIFF) [file pone.0228356.s001.tiff]

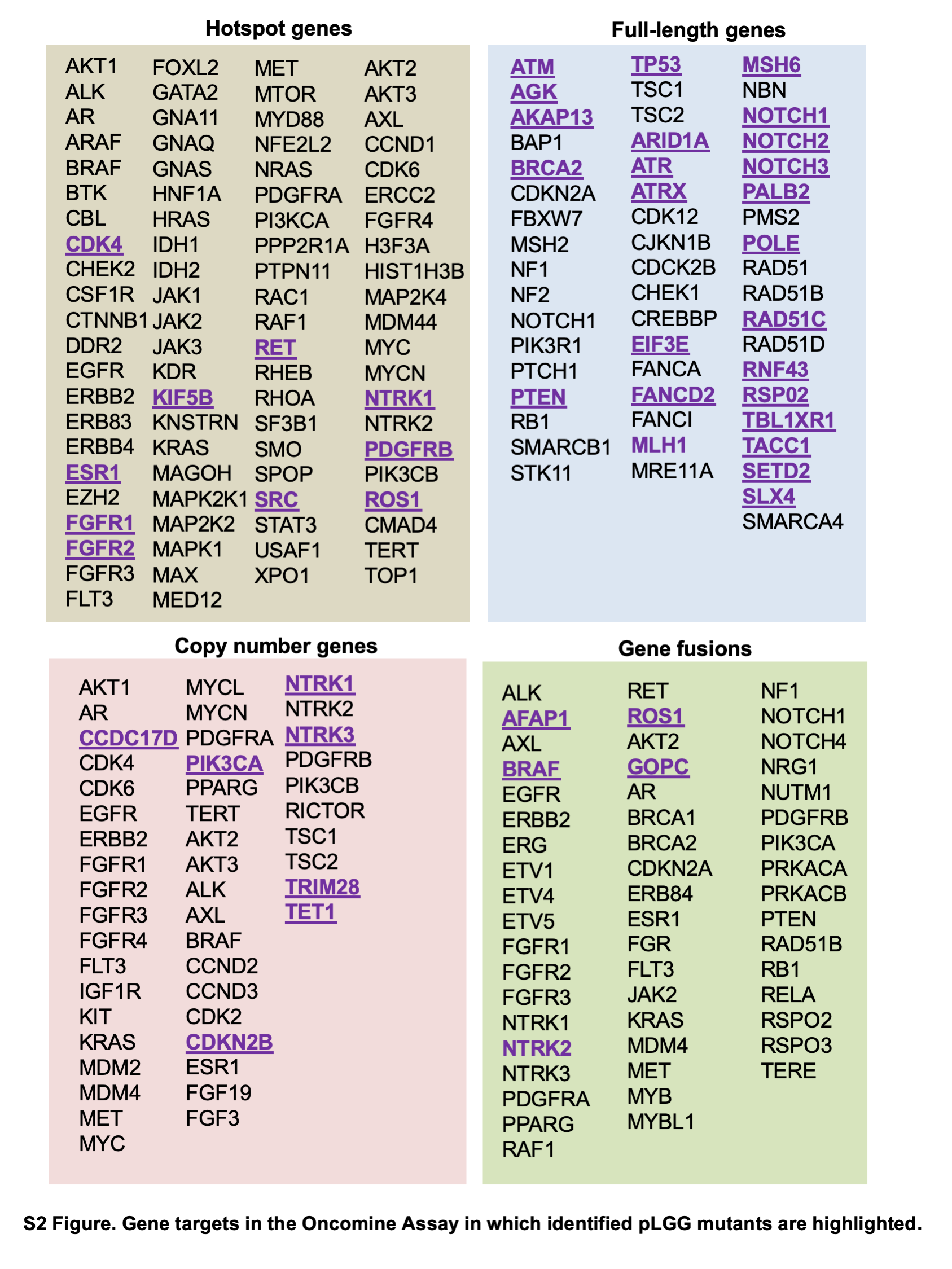

Supplement: S2 Fig — (TIFF) [file pone.0228356.s002.tiff]

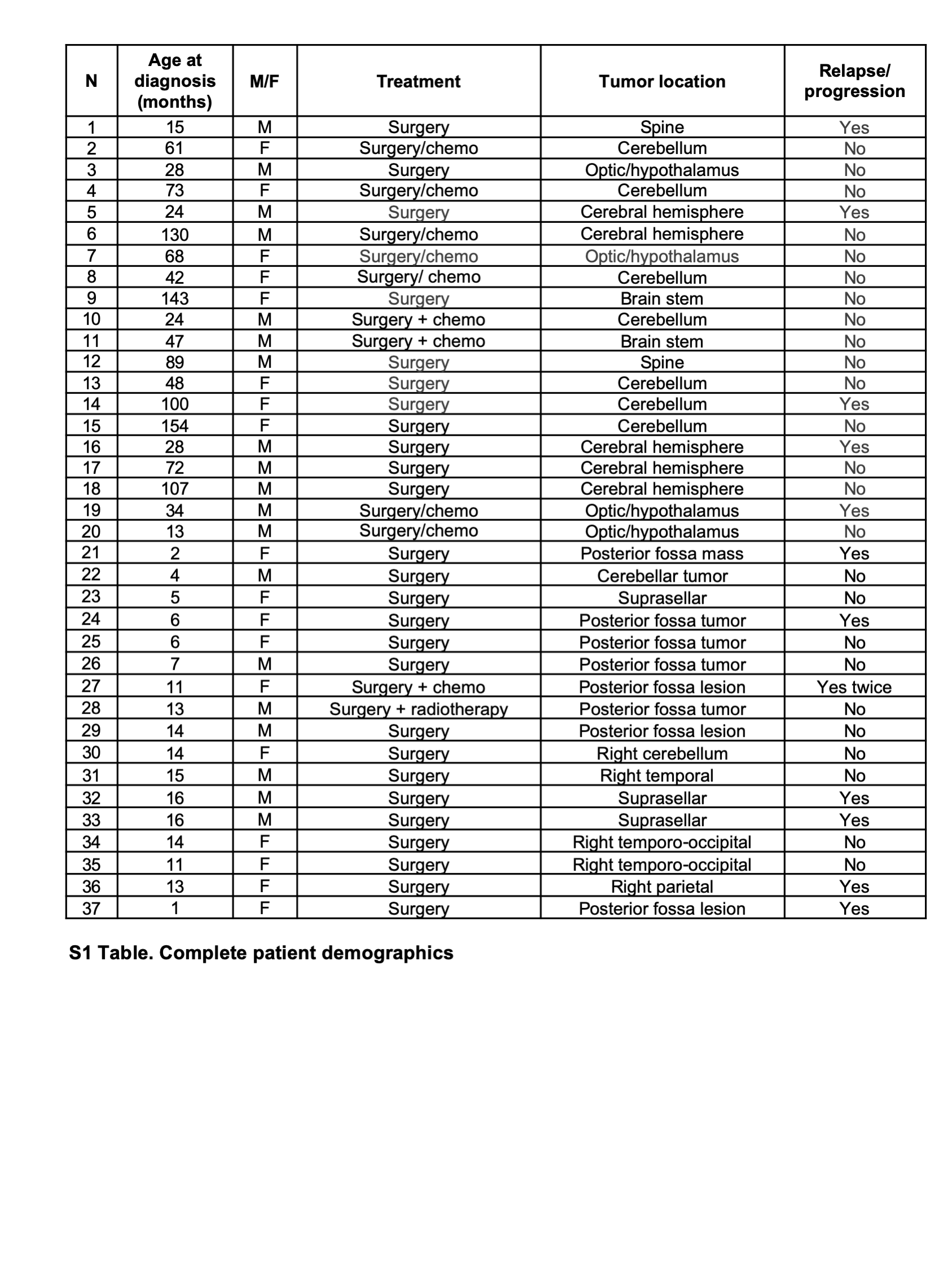

Supplement: S1 Table — (TIFF) [file pone.0228356.s003.tiff]

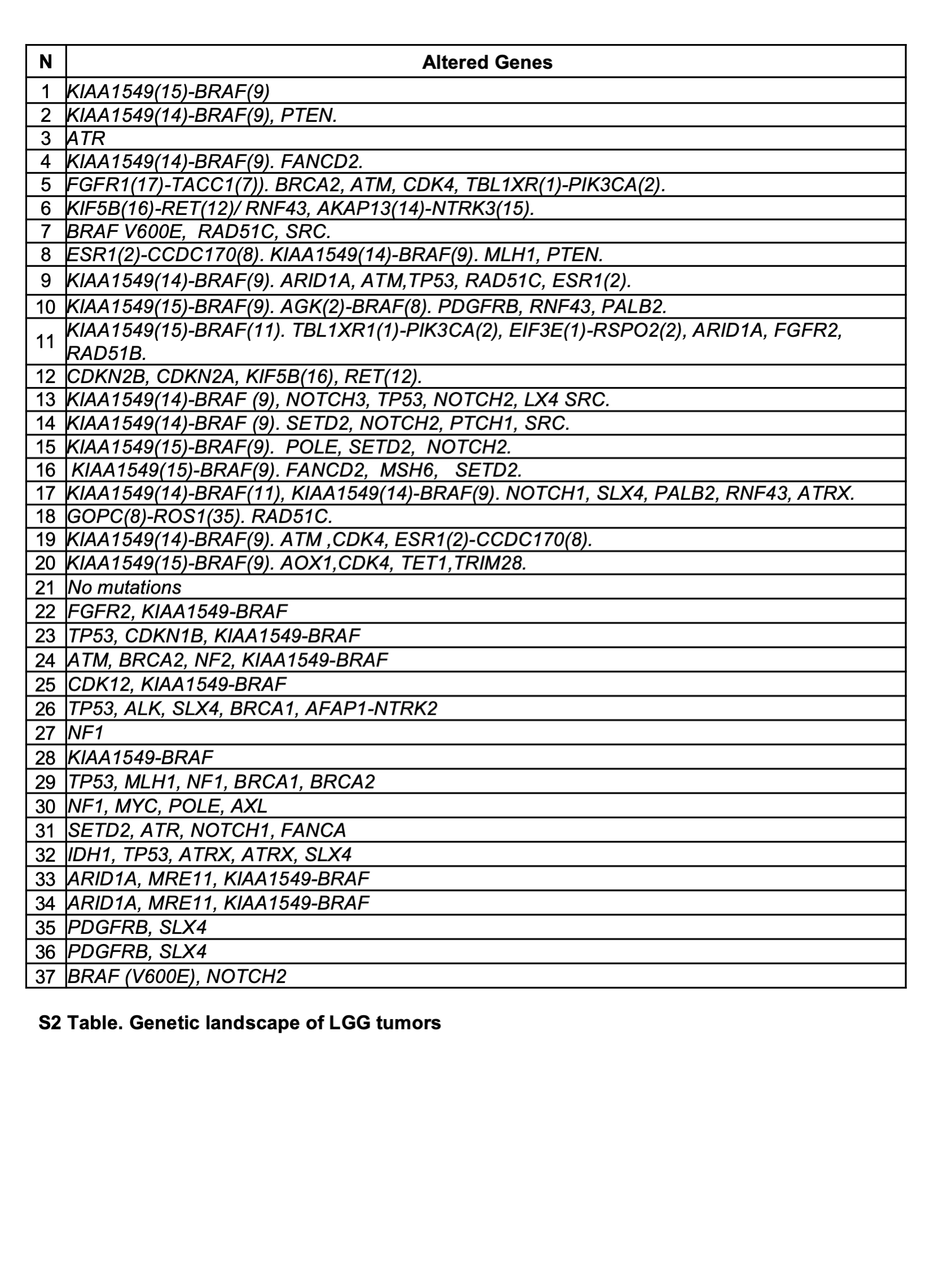

Supplement: S2 Table — (TIFF) [file pone.0228356.s004.tiff]
