# Supplementary material for: Clinical management and genomic profiling of pediatric low-grade gliomas in Saudi Arabia
Source: PLoS One. 2020 Jan 29;15(1):e0228356. doi: 10.1371/journal.pone.0228356 (PMC6988947; doi:10.1371/journal.pone.0228356)
Supplement: Attachment — Submitted filename: ResponseToReviwersLetter-.docx [file pone.0228356.s005.docx]

To the Editor,

We thank you for allowing us to resubmit our article entitled “Clinical management and genomic profiling of paediatric low-grade gliomas in Saudi Arabia” to PLoS one. We thank the reviewers for their kind comments which have been addressed in the point-by-point rebuttal below. We believe we have addressed all concerns of the reviewers and agree that these modifications improve the manuscript. We hope that you review this resubmission favourably.

Yours sincerely

Malak Abedalthagafi, MD

**Reviewer #1:**

(1): Figures 1-3 summarize the clinical, histological, and genetic profiles of the patient base aptly. What is missing however is a display of the data that allows the reader to scan for potential relationships between demographics, clinical metrics, and tumor genotypes? Potential trends in correlations and, just as important, lack of correlations in the metrics are important to report to the literature.

**Response: We thank you for this comment. The demographics and clinical metrics were descried in supplementary Table 1.**

(2) The abstract indicates that an 8-year old patient lacking BRAF alterations underwent gross total resection followed, chemotherapy and hematopoietic stem cells, and remained disease free. In the results section (Lines 182-197), a 4 year old Paediatric High Grade Glioma (pHGG) patient with the GOPC-ROS1 fusion mutation received the same treatment and achieved the same outcome (previously reported in reference 39), whereas an 8-year old patient with the GOPC-ROS1 fusion mutation received a gross resection with no adjuvant chemotherapy. This passage, the abstract, or both need clarification.

**Response: We apologise for the confusion. The treatment regimens of our patient have been modified and corrected for clarity. We thank you for this comment.**

(3) It is inappropriate to describe the NGS-based genotyping approach taken in this study as a genomic approach. A targeted genetic screen of a panel of cancer-related genes does not constitute a genomic profile. The reviewer requests that the manuscript text is edited accordingly.

**Response: We thank you for the comment. This has been modified throughout the manuscript as correctly requested.**

**Reviewer #2:**

1. They distinguish cerebellar tumors from posterior fossa tumors in the figure and text. This is confusing since a cerebellar tumor is by definition a post fossa tumor. This should be clarified.

**Response: Thank you for the comment. This has now been combined as cerebellum/posterior fossa as correctly suggested.**

2. Figure 1 bar graphs of type of surgery, and relapse are unnecessary. This data could be provided in a table.

**These data have been shown as a Table in the modified Figure as requested.**

3. They described one patient whom they treated with radiation - it would be nice if they could give the rationale for radiation in that case.

**We thank you for the comment. Surgery did not provide complete resection so radiotherapy was employed this posterior fossa tumor. We have included this text to the manuscript for clarity.**

4. They include a case of a patient with GOPC-ROS1 fusion pHGG. Why was this included in the series, if this series was about LGG? If they thought it was misclassified, why was it misclassified?

**We apologise for the confusion. Our case was classified as pLGG . The pHGG discussions refer to the GOPC (FIG)-ROS1 fusion in a paediatric high-grade glioma survivor [reference 39 in the manuscript]. We have polished the text describing the treatment regimen of our pLGG patient for clarity.**
